# Determining intrafractional prostate motion using four dimensional ultrasound system

**DOI:** 10.1186/s12885-016-2533-5

**Published:** 2016-07-15

**Authors:** Mariwan Baker, Claus F. Behrens

**Affiliations:** Department of Oncology, Radiotherapy Research Unit, Herlev Hospital, University of Copenhagen, Herlev, Denmark; Center for Fast Ultrasound Imaging, Department of Electrical Engineering, Technical University of Denmark, DK-2800 Lyngby, Denmark; Center for Nuclear Technologies, Technical University of Denmark, DTU Risø Campus, Roskilde, Denmark

## Abstract

**Background:**

In prostate radiotherapy, it is essential that the prostate position is within the planned volume during the treatment delivery. The aim of this study is to investigate whether intrafractional motion of the prostate is of clinical consequence, using a novel 4D autoscan ultrasound probe.

**Methods:**

Ten prostate patients were ultrasound (US) scanned at the time of CT imaging and once a week during their course of radiotherapy treatment in an ethics-approved study, using the transperineal Clarity autoscan system (Clarity®, Elekta Inc., Stockholm, Sweden). At each US scanning session (fraction) the prostate was monitored for 2 to 2.5 min, a typical beam-on time to deliver a RapidArc® radiotherapy fraction. The patients were instructed to remain motionless in supine position throughout the US scans. They were also requested to comply with a bladder-filling protocol. In total, 51 monitoring curves were acquired. Data of the prostate motion in three orthogonal directions were analyzed. Finally, the BMI value was calculated to investigate correlation between BMI and the extent of prostate displacement.

**Results:**

The patients were cooperative, despite extra time for applying the TPUS scan. The mean (±1SD) of the maximal intrafractional displacements were [mm]; I(+)/S: (0.2 ± 0.9); L(+)/R: (−0.2 ± 0.8); and A(+)/P: (−0.2 ± 1.1), respectively. The largest displacement was 2.8 mm in the posterior direction. The percentage of fractions with displacements larger than 2.0 mm was 4 %, 2 %, and 10 % in the IS, LR, and AP directions, respectively. The mean of the maximal intrafractional Euclidean distance (3D vector) was 0.9 ± 0.6 mm. For 12 % of the fractions the maximal 3D vector displacements were larger than 2.0 mm. At only two fractions (4 %) displacements larger than 3.0 mm were observed. There was no correlation between BMI and the extent of the prostate displacement.

**Conclusions:**

The prostate intrafractional displacement is of no clinically consequence for treatment times in the order of 2 – 2.5 min, which is typical for a RapidArc radiotherapy fraction. However, prostate motion should be considered for longer treatment times eg if applying conventional or IMRT radiotherapy.

## Background

In external beam radiotherapy of the prostate, it is essential that the prescribed dose is precisely delivered to the prostate, while reducing toxicity to rectum and bladder. This can be achieved by daily accurate positional verification of the prostate using image-guided radiation therapy (IGRT) [1, 2]. Since variation in prostate position during treatment delivery (intrafractional prostate motion) might occur, the target may be underdosed due to a possible prostate shift [3, 4]. Bladder filling variations, rectal volume changes, and respiratory motion are some of the factors that might lead to intrafractional prostate motion [5–7].

To determine the intrafractional prostate motion, various imaging techniques have been investigated in different studies, such as X-ray imaging, kilovoltage (kV) 3D CBCT and megavoltage (MV) imaging, Cine-MRI, in-room CT, implanted markers and transponders, and ultrasound [8–11]. Most of the studies are based on acquiring pre- and post-treatment images [12, 13].

Various techniques are developed to enable real-time online prostate localization and monitoring, such as: tracking implanted electromagnetic transponders (Calypso Medical Technologies, Seattle, WA), and tracking fiducial markers (FMs) or implanted radioactive seeds (in Brachytherapy) using real-time X-ray imaging. Recently, transperineal ultrasound (TPUS) autoscan (Clarity®, Elekta Inc., Stockholm, Sweden), a non-ionizing, non-invasive imaging modality, has been developed to allow real-time prostate tracking [14]. Various studies have investigated the Calypso system to determine intrafractional prostate motion [15–19], however, to our knowledge, there is only one study (Ballhausen et al.) using the Clarity TPUS system [20]. In the study by Ballhausen et al. data from 6 prostate patients are investigated. Ballhausen et al. concludes that “intrafraction motion of the prostate is a random walk” and the prostate moves away from the isocenter during treatment delivery. In the present study, the TPUS system was utilized to determine the intrafractional motion of the prostate.

Using advanced radiotherapy techniques, such as volumetric modulated arc therapy (VMAT) or RapidArc® Radiotherapy Technology, the treatment delivery time can be significantly reduced, thus minimizing intrafractional prostate motion [21, 22]. In our institution, RapidArc radiotherapy is a standard technique for treating prostate cancer patients, and a typical beam-on time for a treatment fraction is approximately 2.5 min.

The aim of this study is to investigate whether the intrafractional prostate motion, during a time interval corresponding to the beam-on time for RapidArc, is within 2.0 mm. 2 mm is a tolerance value which is perceived to be clinically irrelevant according to the British Ionization Radiation Medical Exposure Regulations 2000 (IRMER 2000).

## Methods

### Patients

Ten prostate cancer patients, with an average age of 68 years (range 58–76 years), were US scanned in the CT room and once a week in the treatment room utilizing the Clarity 4D TPUS monitoring system. All post prostatectomy patients were excluded. The patients received a cumulative dose of 76 Gy in 38 fractions, 2.0 Gy per fraction, 5 fractions per week. Table 1. comprises patient specifics, US scans, and the treatment technique. The TPUS scans were performed by six radiation therapy technologists (RTTs). The study was approved by the Danish national ethical committee, and voluntary informed consent was obtained for each participant according to the World Medical Association Declaration of Helsinki (1975/2000). The patients were placed in a supine position and instructed to remain motionless throughout the subsequent scans. To ensure better image quality, the patients were requested to comply with a moderate bladder-filling protocol.

**Table 1:** Patients characteristics, ultrasound scans, and treatment technique

Age (Y)	
Mean	68
Range	58–76
BMI	
Mean	27
Range	22–37
PSA level, ng/mL	
Mean	12.4
Range	2–22
Differentiation (Gleason Score)	
Mean	5.7
Range	2.4–8.8
Fractions	
Total fractions	38
Dose/fraction [Gy]	2.0
Race/ ethnicity	Danish patients
Prostate patients	Excluding all post prostatectomy
Radiation treatment	Only external radiotherapy (RapidArc)
Bladder filling	A standard bladder filling protocol
Number of US scans/patient	4–6 scans
Total number of US scans	51 scans

### Clarity ultrasound system

The Clarity system consists of two mobile units (one in the CT room and the second in the treatment room), which are connected through a workstation/server. The workstation was used for target delineation and retrieving the prostate monitoring curves. Details of US 3D image reconstructions and the precision of the system are explained thoroughly in a previous study [23]. In short, a ceiling-mounted infrared (IR) camera recognizes the US probe by detecting the IR-reflectors affixed to them. This is essential for determining the geographical position of the reconstructed anatomical structures.

To enable superimposition of the reconstructed US images from the 4D autoscan probe on the CT images, the system was calibrated to the same coordinate system as the CT simulation and treatment rooms. The calibration procedure was accomplished by means of a dedicated alignment phantom provided by the vendor.

### CT room

All patients underwent a treatment planning CT scan followed by an MRI scan. After the CT-scan the 3D-TPUS scan was acquired. The patient was instructed to remain still during image acquisition and US monitoring. Afterwards, the US-CT fusion was performed, on which the prostate volume was delineated and used as a reference for weekly US imaging in the treatment room.

### Autoscan probe

The autoscan-US probe consists of a one dimensional (1D) transducer array of 128 elements, using a central frequency of 5.0 MHz. The probe is provided with IR reflectors fixed in a way that can be detected by the ceiling mounted IR-camera. Initially, the probe is affixed to a TPUS kit, placed over the coach under the patients’ knees, and pushed gently to the patient for scanning. The motorized head is capable of real-time online scanning of the prostate.

### Prostate monitoring in the treatment room

The patient was prepared for daily treatment by aligning him to the lasers guided by reference marks (tattoos) on the skin, ie to reproduce the patient’s setup position from the CT-simulation room. Prior to treatment, the inter-fraction patient positioning was corrected for by daily kV images by utilizing three implanted FMs. Once a week and immediately after treatment delivery the TPUS monitoring system was set up for real-time tracking of the prostate. The time dependent prostate displacements (prostate intrafractional motion) were recorded in 2 to 2.5 min. For each patient, 4–6 US scans were acquired. The prostate displacements in the three orthogonal directions were recorded for retrospective analysis.

### Statistical analysis

The prostate data, showing real-time prostate COM position in the Inferior-Superior (I/S), Left-Right (L/R) and Anterior-Posterior (A/P) directions, were evaluated using the Clarity workstation by one observer (MB). For some fractions, irregular fluctuating data at the beginning of the monitoring curve were discarded. The maximum prostate displacement in each direction was recorded for each fraction. Furthermore, the Euclidean distance (3D vector) was computed and the maximal 3D vector was recorded for each fraction. Finally, the mean (±1 standard deviation (SD)) of the maximal displacements in each direction and for the 3D vector were calculated. The BMI value for each patient was calculated to investigate correlation between magnitudes of the displacement against the BMI. For the data and statistical analysis the statistical program R (version 2.15.3) was used.

## Results

All the patients enrolled in this study were cooperative, despite the extra time of about 10 min for each fraction needed for the autoscan setup and prostate monitoring. None of the patients expressed any discomfort during the autoscan setup, nor from the TPUS real-time scanning.

Data analysis of the 51 TPUS scans resulted in mean values (±1SD) of the maximal intrafractional displacements of the prostate [mm]; I(+)/S: (0.2 ± 0.9); L(+)/R: (−0.2 ± 0.8); and A(+)/P: (−0.2 ± 1.1). The largest displacement was 2.8 mm in the posterior direction. The percentage of fractions larger than 2.0 mm was 4 %, 2 %, and 10 % in the IS, LR, and AP directions, respectively. The mean Euclidean distance of the 51 scans was 0.9 ± 0.6 mm. The percentage of fractions with 3D vector displacement larger than 2.0 mm was 12 %, and only 2 scans (4 %) showed displacements larger than 3.0 mm. No correlation was found between BMI-value and the magnitude of the prostate displacement.

**Fig. 1:**
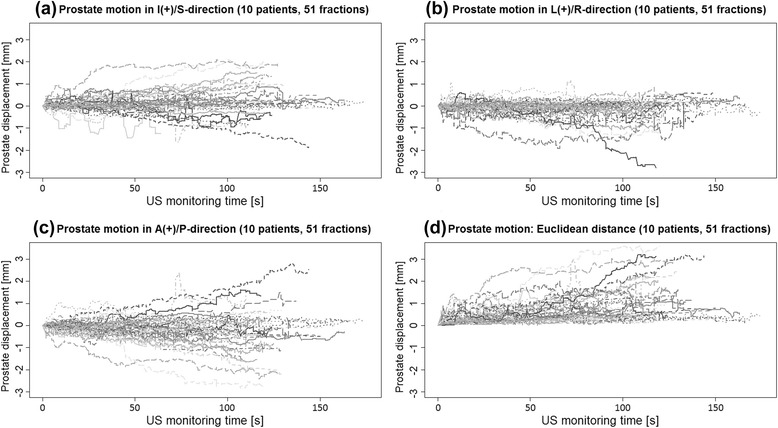
Intrafractional prostate displacement; (**a-c**) Monitoring graphs show the prostate motion for the total 51 US scans in I/S, L/R, and A/P directions, (**d**) The 3D vector displacement of the prostate center of mass

The monitoring curves of the prostate show variations in prostate displacement between different fractions (Fig. 1). A common zero point, start point for the curves, was established by choosing an arbitrary position-value for each scan/curve, a position immediately after the prostate tracking initiated, and then subtracting that value from the rest. For some fractions, the tendency of larger intrafractional displacement for prolonged observation times can be observed. The figure also indicates that the extent of the prostate intrafractional displacement has its largest dispersal in the A/P direction, and least displacements in the L/R direction.

**Fig. 2:**
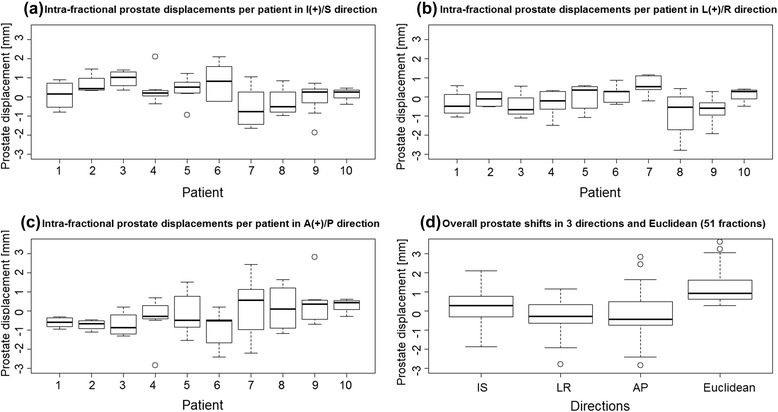
Boxplots of the intrafractional prostate displacements; (**a-c**) Boxplots of the prostate displacements for ten patients in I/S, L/R, and A/P directions. (**d**) Boxplot of overall prostate displacements in I/S, L/R, and A/P directions, including Euclidean (3D vector) distance

Figure 2a-c presents boxplots of the maximal displacements at each fraction for each of the ten patients in I/S, L/R, and A/P directions, respectively. The horizontal band inside the box indicates the second quartile (median), the lower and the upper edges of the box indicate the first (25^th^) and third (75^th^) quartiles. Furthermore, the lower and the upper extremes of the whiskers display the minimum and maximum values in the absence of single data point outliers. The individual boxplot of maximal prostate displacements is based on analysis of 4–6 TPUS scans per patient. As can be observed, there are some variations in prostate displacements between the patients and in the different directions. The overall maximal prostate displacement in all three directions and the Euclidean 3D vector were shown to be less than 2.0 mm for most of the fractions (Fig. 2d).

## Discussion

Intrafractional prostate motion has been investigated previously using pre- and post-treatment imaging. One weakness in applying this method is inter-observer matching uncertainty [24]. Despite matching variability, using portal imaging of seeds, there is an additional error associated with the thickness of the CT-slices that may introduce further uncertainty into the determination of the displacement. Another drawback is that the prostate can be displaced and then revert to its initial position, as can be observed in Fig. 1, which cannot be detected by only a single snapshot pre- and post-treatment image [25]. Therefore real-time tracking is a proper monitoring method to accurately detect intrafractional prostate motion. Table 2 tabulates the results of the present study and previously reported data

**Table 2:** Intrafraction prostate motion in three directions and 3D vector; a comparison of the present study with previously published data using different systems

				Mean of max prostate shifts (±1SD) [mm]				
Investigator, year	N (fractions)	System	Time [min]	I(+)/S	L(+)/R	A(+)/P	% 3D vector shift	3D-vector [mm]
Lometti et al., 2005 [33]	11 (133)	*MV fluoroscopy: real-time FM tracking*	1				>2 mm 4 %	0.7 ± 0.5
Li HS et al., 2008 [4]	35 (1267)	*Calypso*	12	0.3 ± 0.7	0.0 ± 0.3	0.4 ± 0.6		
Kupelian et al., 2008 [2]	17 (550)	*Calypso*	10				>3 mm 14 %	
Langen et al., 2008 [18]	17 (550)	*Calypso*	2				>3 mm 3 %	
Li et al., 2009 [19]	20 (157)	*Calypso*	11.4				>3 mm 19 %	
Vargas et al., 2010 [29]	7 (68)	*Cine-MRI*	4					0.41 ± 1.2
Wang et al., 2011 [34]	29 (1061)	*Calypso*	3				>3 mm 9 %	
Smeenk et al., 2011 [30]	15 (576)	*Calypso*	2.5				>3 mm 1.4 %	
Ng et al., 2012 [32]	10 (268)	*Real-time kilovoltage FM tracking*	3–4				>3 mm 5.6 %	
Mayyas et al., 2013 [9]	8	*Pre- post-treatment kV images*		0.8 ± 2.7	0.2. ± 2.1	−0.3 ± 2.4		
Mayyas et al., 2013 [9]	19	*Calypso*		0.0 ± 1.5	0.0 ± 0.6	0.0 ± 1.3		
Tong et al., 2015 [8]	236 (8660)	*Calypso*	2				>2 mm 13 %	
Choi et al., 2015 [28]	12 (336)	*Transrectal US/3 FMs*	4–5	0.6 ± 0.6	−0.3 ± 0.3	−0.7 ± 0.6	>2 mm 11 %	1.1 ± 0.8
Present study	10 (51)	*Clarity real-time TPUS*	2–2.5	0.2 ± 0.9	−0.2 ± 0.8	−0.2 ± 1.1	>2 mm 12 %	0.9 ± 0.6

Intrafractional motion of prostate patients (N) in 3D vector, I/S, inferior-superior, L/R, left-right, and A/P, anterior-posterior directions, using TPUS, transperineal Clarity ultrasound (US) system, Calypso system, implanted fiducial markers (FMs), CINE-MRI, and endorectal balloon

.

In this study the 4D TPUS monitoring system was shown to be able to track the prostate. The 4D autoscan probe have previously been applied as a reliable tool to measure the prostate displacement during transabdominal probe simulation [26]. The operators, with experience from frequently used transabdominal scanning, reported that using the TPUS system, it was easier to acquire images and identify soft tissue structures compared to transabdominal scanning. In a previous phantom study, Abramowitz et al. found good agreement between the TPUS autoscan and Calypso system (Varian Medical Systems, Palo Alto, CA) in tracking the embedded prostate-like sphere [27]. The Calypso system has been investigated in different studies, which have confirmed that it is an accurate monitoring method for tracking the prostate gland.

In the current study, prostate monitoring was limited to approximately 2.5 min, a typical beam-on time to deliver a RapidArc fraction. We observed that the prostate is not stationary during the tracking time, and displacements tend to increases with the elapsed monitoring time, which is in line with the findings of other published papers [20]. Ballhausen et al. found that the intrafractional motion of the prostate tends to “a linear increase of the variance” with the duration of the fraction. Furthermore, we found that the displacement varies for different fractions and also for different patients.

In our study, we noticed that, for most of the fractions, the intrafractional prostate motion was mostly smaller than 2 mm, for a period of 2 to 2.5 min. Comparably, Choi et al. [28], using transrectal ultrasound scans to track three implanted fiducial markers (12 patients and 336 fractions), observed that only 11 % of the 3D vector displacements were larger than 2 mm during 4–5 min of monitoring (Table 2). Similarly, Li et al. [4], utilizing a Calypso tracking system (35 patients and 1267 fractions), reported that the intrafractional motion of the prostate is mostly less than 2 mm, especially for the first three minutes of tracking. They concluded that a CTV-PTV margin of 2 mm is adequate to ensure covering the target with the planned prescribed dose, despite larger intrafractional uncertainty for some of the patients. Furthermore, Vargas et al. [29], in an Cine-MRI study, calculated the CTV-PTV margin to be 2.1 mm, comparable to Li et al.’s, which would account for the intrafractional motion. They showed also that the prostate motion is smaller in supine than prone patient positioning. On the other hand, Smeenk et al. [30], again using Calypso system (15 patients and 576 fractions), discovered that prostate motion in 3D vector was larger than 3 mm in only 1.4 % of the fractions during the first 2.5 min, but increased to 18 % for a monitoring duration of 10 min. Comparably, Tong et al. [8], also using Calypso tracking system (236 patients and 8660 fractions), showed that up to 20 % of the prostate shift can be larger than 3 mm when tracking time is 12 min. But they ascertained that the prostate motion, for the first three minutes, is very small (<2 mm) for most patients. Similarly, Shelton et al. [31] investigated the intrafraction prostate motion, once again using Calypso system (37 patients and 1332 fractions), and found that the overall mean value of the 3D vector displacement is less than 3 mm for the first 3 min, but increased with duration time to up to 6.5 mm in the fourteenth minute. Likewise, Kupelian et al. [2], once more using Calypso system (17 patients and 550 fractions) detected that during 10 min monitoring in 14 % of the fractions the shift was larger than 3 mm. Furthermore, Ng et al. [32], in a study of real-time FM tracking, observed that in 62 % of the fractions (10 patients, 268 fractions) the 3D vector displacement of the prostate, during 3–4 min tracking was smaller than 1 mm, and corresponded values were 95 %, 82 %, and 80 % in the LR, IS, and AP directions, respectively. They discovered that only 6 % of the shifts were larger than 3 mm. Moreover, Langen et al. [18], as well using Calypso system (17 patients and 550 fractions), spotted that only 3 % of the shift is larger than 3 mm while tracking the prostate for about 2 min. In addition, Lometti et al. [33], using MV fluoroscopy; real-time FM tracking (11 patients and 133 fractions), confirmed that in only 4 % of the fractions the displacement is larger than 2 mm while tracking for only one minute. Last, Mayyas et al. [9], in a comparison study of four different systems (27 patients and 1100 fractions), found that the percentage of intrafractional shifts within ±1.5 mm was 76 %, 94 %, and 82 % in the I/S, L/R, and A/P directions. In summary, the published evidence in combination with this study justifies the assertion that the intrafractional motion of the prostate is insignificant during the first 2.5 min, thus not of clinical consequence while employing advanced VMAT/ RapidArc.

As all other studies, there are limiting factors in the current study; Firstly, the study does not reveal the magnitude of the intrafractional prostate rotation or form deformation. Second, the number of patients and fractions are limited. Third, a dosimetric study is necessary to investigate the impact of the larger prostate displacements on the clinical treatment outcome of the treatment.

## Conclusion

Intrafractional prostate displacement varies between fractions and for different patients. The displacement is insignificant for the first 2–2.5 min of the monitoring time, but increases with elapsed time. Consequently, prostate shifts during conventional and IMRT treatment delivery should be taken into consideration, but are clinically inconsequential while employing advanced VMAT/ RapidArc techniques.

## Abbreviations

3D, Three dimensional; 4D, Four dimensional; A/P, Anterior-posterior; CT, Computed tomography; CTV, Clinical target volume; FM, Fiducial marker; I/S, Inferior-superior; IGRT, Image-guided radiation therapy; IMRT, Intensity modulated radiation therapy; kV, Kilo-voltage; L/R, Left-right; MHz, Mega hertz; MRI, Magnetic resonance imaging; MV, Mega-voltage; PTV, Planning target volume; RTT, Radiation therapy technologist; SD, Standard deviations; TPUS, Transperineal ultrasound; US, Ultrasound; VMAT, Volumetric modulated arc therapy
